# Dr. Frederick G. Trowbridge

**Published:** 1911-11

**Authors:** 


Dr. Frederick G. Trowbridge, a classmate of the former, died at Watertown, N. Y., of the same cause, September 20, 1911, aged 53.

